# Rupture du tendon d’Achille

**DOI:** 10.11604/pamj.2020.37.113.25339

**Published:** 2020-10-02

**Authors:** Maher Teka, Hazem Ben Ghozlen

**Affiliations:** 1 Service de Chirurgie Orthopédique, Hôpital Taher Sfar, Mahdia, Tunisie

**Keywords:** tendon d’Achille, chirurgie, rupture, Achille’s tendon, surgery, rupture

## Abstract

Achille’s tendon rupture (A, B) involves a tendon with degenerative lesions that are asymptomatic until rupture. These degenerative lesions result from tendon microtraumas and overexertion almost always related to sport and hyperactivity as well as to tendon aging. Etiologic research is necessary in patients with Achille’s tendon rupture. Favoring factors include morphological disorders (hollow foot; unequal length) while triggering factors include insufficient training, running on solid ground, poor shoe conditions. We here report the case of a 50-year old female patient with no particular past medical history with left Achille’s tendon rupture (A) surgically treated (C, D) by Bosworth technique based on plaster-cast immobilization using plaster cast boot for 21 days. After two months of rehabilitation, functional recovery was complete. However, the etiological assessment showed early-stage systemic lupus erythematosus.

## Image en médecine

*La rupture du tendon d’Achille (A, B) survient sur un tendon atteint de lésions dégénératives asymptomatiques jusqu’à la survenue de la rupture. Ces lésions dégénératives sont le résultat des microtraumatismes et surmenage tendineux liés presque toujours au sport et à l’hyperactivité ainsi qu’au vieillissement du tendon. Une recherche étiologique est nécessaire devant toute rupture du tendon d’Achille. On peut trouver des facteurs favorisants tels que des troubles morphologiques (pied creux; inégalité de longueur) ou des facteurs déclenchants tels que l’insuffisance d’entraînement, course sur terrain solide, mauvais état des chaussures. Nous rapportons le cas d’une patiente âgée de 50 ans, sans antécédents pathologiques ayant présenté une rupture du tendon d’Achille gauche (A) qui a été traité chirurgicalement (C, D) selon la technique de Bosworth avec immobilisation par plâtre cruro-pédieux pendant 21 jours relayés par une botte plâtrée pendant la même période. Après deux mois de rééducation la récupération fonctionnelle était complète. Cependant, le bilan étiologique a conclu à un lupus érythémateux disséminé dans sa forme débutante*.

**Figure 1:**
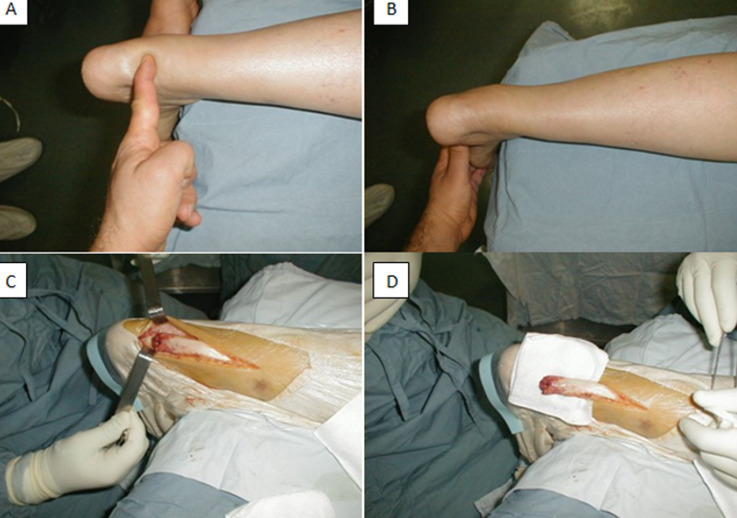
rupture du tendon d’Achille, aspect pré et peropératoire (A,B,C,D)

